# High-throughput three-dimensional chemotactic assays reveal steepness-dependent complexity in neuronal sensation to molecular gradients

**DOI:** 10.1038/s41467-018-07186-x

**Published:** 2018-11-12

**Authors:** Zhen Xu, Peilin Fang, Bingzhe Xu, Yufeng Lu, Jinghui Xiong, Feng Gao, Xin Wang, Jun Fan, Peng Shi

**Affiliations:** 1 Department of Biomedical Engineering, City University of Hong Kong, 83 Tat Chee Ave, Kowloon, Hong Kong SAR, 999077 China; 2 Department of Material Science and Engineering, City University of Hong Kong, 83 Tat Chee Ave, Kowloon, Hong Kong SAR, 999077 China; 3 Department of Biomedical Science, City University of Hong Kong, 83 Tat Chee Ave, Kowloon, Hong Kong SAR, China; 4 Shenzhen Research Institute, City University of Hong Kong, 518000 Shenzhen, China

## Abstract

Many cellular programs of neural development are under combinatorial regulation by different chemoattractive or chemorepulsive factors. Here, we describe a microfluidic platform that utilizes well-controlled three-dimensional (3D) diffusion to generate molecular gradients of varied steepness in a large array of hydrogel cylinders, allowing high-throughput 3D chemotactic assays for mechanistic dissection of steepness-dependent neuronal chemotaxis. Using this platform, we examine neuronal sensitivity to the steepness of gradient composed of netrin-1, nerve growth factor, or semaphorin3A (Sema3A) proteins, and reveal dramatic diversity and complexity in the associated chemotactic regulation of neuronal development. Particularly for Sema3A, we find that serine/threonine kinase-11 and glycogen synthase kinase-3 signaling pathways are differentially involved in steepness-dependent chemotactic regulation of coordinated neurite repellence and neuronal migration. These results provide insights to the critical role of gradient steepness in neuronal chemotaxis, and also prove the technique as an expandable platform for studying other chemoresponsive cellular systems.

## Abstract (web summary)

In vitro chemotactic assays are often 2D, low-throughput and lack fine gradient control. Here the authors present a hydrogel-based microfluidic platform for high-throughput 3D chemotactic assays, and use it to study neuronal sensitivity to guidance molecule gradient steepness.

## Introduction

Cell migration and neurite projection are key cellular processes in the development of the nervous system^1–3^. In an extremely precise format, progenitor neurons migrate to targeted coordinates from different origins and elaborate extensive neurite outgrowth to allow the wiring of brain circuits^2^. These processes are regulated by the graded distribution of diffusive or substrate-bounded guidance cues or chemotaxis^1,4^. Although there has been great success in determining the identity of various chemotactic molecules, such as netrin^5^, semaphorin (Sema)^6^, slit proteins^7^, ephrin^8^, and neurotrophin factors^9^, our understanding about many details of neuronal chemotaxis is still in its early stages^10^. Some molecules employ a concentration-dependent mechanism to regulate neurite extension^11,12^. Gradients with different steepness could also induce distinct responsive mode in growing axons^13,14^. It has also been observed that certain types of neurons can migrate with simultaneous extension of axons in the opposite direction^3,15^. These reports suggest the existence of additional and unresolved complexity in neuronal chemosensation. In addition, some molecules are suggested to play shared roles in the guidance of migrating neurons and axonal projection^16,17^, but little has been done to elucidate the integration of the two cellular programs within individual cells. In fact, many important questions to neuronal chemotaxis remain largely unexplored, essentially due to a lack of experimental tools that can accurately control the spatial and temporal profile of the molecular gradient for system-level investigations.

In the past few decades, many guidance molecules have been discovered and studied using in vitro chemotactic assays due to the difficulty of characterizing the exact profile of molecular gradient in vivo. Trans-well assays are usually used to measure the migration capability of cultured neurons^18^. Cocultures of commissural axons with floor plate cells enabled direct visualization of neurite guidance by secreted netrin-1^19^. Micropipette perfusion and stripe assays played an instrumental role in the discovery of novel axonal guidance molecules^9,20^. These assays are mostly limited to two-dimensional (2D) cultures and lack sophisticated gradient control or the throughput required for systematic studies^10,21^. Recently, some microdevice-based assays were developed and used to study different aspects of neuronal chemotaxis, including the role of gradient steepness^13,14^, temporal filtering^22^, and growth cone adaption^23^. The convergence of micro-technology and neuroscience research clearly expands the arsenal for advancing our understanding about chemotactic molecular guidance in neurons^24–27^.

In this study, we develop a microfluidic platform that incorporates arrays of Matrigel-cylinders to allow high-throughput generation of a large-scale library of molecular gradients with distinct steepness. When primary neurons were seeded into the hydrogel, a massive array of three-dimensional (3D) neuron cultures were established with each of the cylinders containing a distinct gradient profile. Accordingly, hundreds of 3D chemotactic assays can be performed in parallel to allow quantitative investigation of the steepness-dependent neuronal response associated with both neuronal migration and axonal projection. Using this platform, we systematically studied neurons’ sensitivity to the steepness of three classical guidance molecules, including netrin-1, nerve growth factor (NGF), and Sema3A, and revealed dramatic diversity and complexity in relevant chemotactic regulations. Particularly for Sema3A, we found that (serine/threonine kinase-11) STK11 and (glycogen synthase kinase-3) GSK3 signaling pathways are differentially involved in the gradient steepness-dependent regulation of neurite guidance and neuronal migration, and that GSK3 activity is especially critical for sensing Sema3A steepness in neuronal migration. Collectively, these results provide insights into the role of gradient steepness in neuronal chemotaxis. Also, we believe that our 3D high-throughput chemotactic assay platform (HT-ChemoChip) provides an innovative experimental framework to potentially advance the field of neurobiology.

## Results

### Design of the microfluidic device

**Fig. 1:**
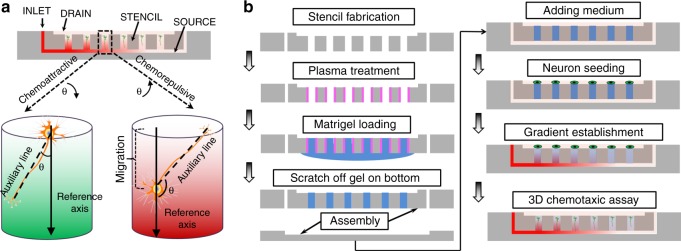
Design of the high-throughput 3D chemotactic assays. **a** Schematic showing the use of suspended microfluidics for generating molecular gradients with different steepness in an array of hydrogel cylinders. The enlarged inset shows the method to quantify neuronal growth, including neurite guidance and soma migration, for cells cultured in the hydrogel cylinders containing chemoattractive or chemorepulsive cues. **b** The step-by-step protocol for device assembly and its application to high-throughput 3D chemotactic assays

As its technical innovation, the microfluidic device relies on a simple diffusion process to establish molecular gradients in a well-designed 3D space. As shown in Fig. 1, the device was ~1 cm in width and ~3 cm in length, and was based on a suspended array of Matrigel cylinders, each of which was measured as 200 µm in diameter and 250 µm in height, and was spaced by 200 µm from the neighboring ones. Each device was composed of three layers: a SOURCE layer, a suspended STENCIL layer (250 µm thick), and a DRAIN layer. The inlet of the bottom SOURCE layer was used to introduce guidance molecules (e.g., netrin-1, NGF and Sema3A). The height of the SOURCE layer was sufficiently small (~400 µm) to ensure that the molecules spread in this layer just by diffusion to establish a horizontal gradient in the *X*–*Y* plane, providing a gradually decreasing input concentration for each Matrigel cylinder within the suspended STENCIL layer. By further diffusion in the vertical direction, various gradients along the *Z*-axis were established within each of the hydrogel cylinders. Because of the large volume of culture medium in the DRAIN layer, the concentration at the top of each cylinder was approximately the same as the background level, which effectively resulted in descending gradient steepness in the hydrogel cylinders when their positions moved away from inlet of the SOURCE layer. When primary hippocampal neurons were cultured in the hydrogel cylinders by directly seeding them in the DRAIN layer, the device could be used to test tens of different gradient steepness (along *X*-axis) with multiple replicates (along *Y*-axis) in a single HT-ChemoChip.

### Characterization of molecular gradient

**Fig. 2:**
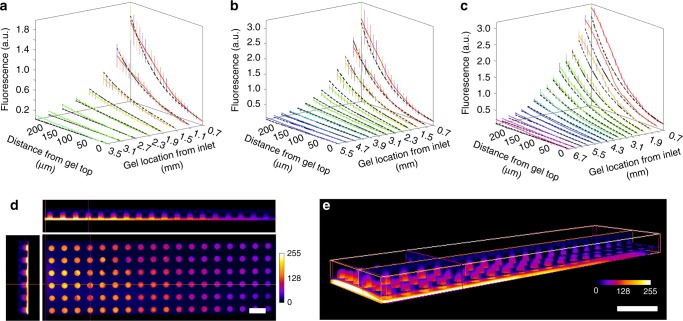
Experimental and computational characterization of gradient generation. **a**–**c** Comparison of the experimental measurements (colored solid lines) and theoretical modeling (black dash lines) of the gradient profiles in the hydrogel cylinders at 7 h (**a**), 12 h (**b**), and 24 h (**c**) after addition of fluorescent dextran, *n* = 3, error bars indicate standard error of the mean. **d** Fluorescent images of different cross-sectional planes showing the 3D distribution of FITC-dextran molecules in the hydrogel cylinders after 12 h diffusion. Scale bar, 500 µm. **e** 3D reconstruction of the confocal scans showing the overall gradient generation in the HT-ChemoChip. Scale bar, 1 mm. Source data are presented as a Source Data file

For an overall evaluation of the gradient generation principle in an HT-ChemoChip, we first performed computational simulation to model the diffusion process and the resulted gradient profiles (Fig. 2a). For 70 kDa dextran molecules, at 7, 12, or 24 h after adding dextran via the inlet of the SOURCE layer, we acquired an array that contained different concentration gradients in the hydrogel cylinders, which was well consistent with our theoretical proposition (Fig. 2a–c). In parallel, we also performed diffusion experiments with fluorescently labeled dextran (70 kDa) to validate and confirm the strategy using confocal microscopy. When the imaging setting (e.g., laser power and scanning speed) was fixed, a linear dependence between the dextran concentrations and fluorescence intensities was achieved (Supplementary Fig. 1), and was used to calculate the actual concentrations from the fluorescence values in later experiments. After the addition of dextran, the molecular concentrations in the hydrogel cylinders were examined at different time points over the following 24 h. The actual molecular concentration profiles in the microfluidic device were reflected by the fluorescence intensities (Fig. 2d) and can be directly visualized by a 3D reconstruction of fluorescence scanning along the *Z*-axis (Fig. 2e). The quantification of the gradient steepness in a hydrogel cylinder was first performed by calculating the slope of a linear-fit for the normalized concentration profile. Furthermore, we also derived a fractional change-based measurement by fitting the concentration profile with an exponential decay curve. Because the gradient was established by taking advantage of 3D diffusion of biological molecules (e.g. netrin-1, sema3A, and NGF) in the HT-ChemoChip, it is expected that the gradient profiles are affected by the diffusion coefficients of different molecules, which is related to their molecular sizes (weights). In our characterization, 70 kDa dextran was used to emulate the diffusion of netrin-1 (67.5 kDa), which generated gradient profiles with a slope ranging from 0.1 × 10^-3^ to 3.7 × 10^-3^ μm^−1^ in hydrogel cylinders of 0.3–7.1 mm from the SOURCE layer inlet, or with a fractional change (over 10 μm) ranging from 2.1% to 9.4% (Supplementary Fig. 2). Similarly, we used 10 kDa and 110 kDa dextran to emulate the diffusion of NGF (13.5 kDa) and Sema3A (110 kDa) respectively, resolving a set of steepness profiles ranging from 0.3 × 10^-3^ to 3.7 × 10^-3^ μm^−1^ (or 3.6–10.4%) for NGF and 1.0 × 10^-5^–3.6 × 10^-3^ μm^−1^ (or 0.6–8.8%) for Sema3A. Notably, this quantification was only an approximate evaluation of the nonlinear concentration curves, but clearly demonstrated the feasibility of the HT-ChemoChip for generating large-scale 3D gradients of different steepness for high-throughput chemotactic assays.

### Application to 3D chemotactic assays

**Fig. 3:**
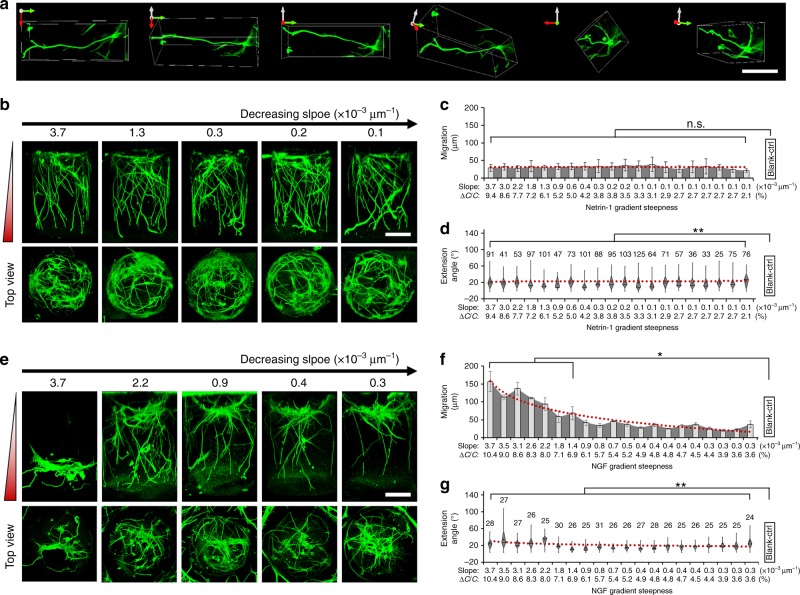
Neuronal response to netrin-1 or NGF gradient of varied steepness. **a** βIII-tubulin fluorescence image of different angles showing the 3D morphology of a single neuron cultured in a hydrogel cylinder, scale bar, 100 µm. **b** Side and top views of 3D cultured hippocampal neurons (βIII-tubulin) in response to netrin-1 gradient of different steepness, scale bar, 100 µm. **c** Quantitative analysis of neuronal migration and related association with netrin-1 gradient steepness, *n* = 4, error bars indicate the standard deviation. **d** Box-plots for quantitative analysis of neurite guidance in response to varied netrin-1 gradient steepness, more than 25 neurites were pooled from four biological replicates. **e** Side and top views of 3D cultured neurons (βIII-tubulin) in response to the NGF gradient of varied steepness, scale bar, 100 µm. **f** Quantitative analysis of neuronal migration and related association with NGF gradient steepness, *n* = 4, error bars indicate the standard deviation. **g** Box plots for quantitative analysis of neurite guidance in response to varied NGF gradient steepness, more than 20 neurites (as indicated on top of each box) were pooled from four biological replicates. For **d**, **g**, the parts of the box indicate 25, 50 and 75 percentiles, and the whiskers indicate 5% and 95%. The square mark indicates mean of the data. For **c**, **d**, **f**, **g**, the red line indicates a logarithmic fitting of the mean. The neuronal growth pattern in each hydrogel cylinder was compared in pairwise to experiments with exactly the same configurations but without any chemotactic factor treatment (Blank-ctrl, Supplementary Fig. 6), * or **indicates a *p*-value <0.05 or <0.005 by paired Kruskal–Wallis tests

Because of a 3D configuration, the HT-ChemoChip provides the capability to characterize neuronal growth with all 3D spatial information (Fig. 3a, Supplementary Movie 1), which is more preferable for recapitulating the in vivo conditions than many existing in vitro chemotactic assays performed using a 2D or cell-aggregate-based culture systems. To systematically demonstrate the utility of the HT-ChemoChip for investigating the role of gradient steepness in neuronal chemotaxis, we firstly tested two classical chemoattractive cues for neuronal development, netrin-1 and NGF, using our 3D high-throughput assays^28^. For netrin-1, neurons were first cultured in the device for 24 h, and 5 ng protein was added to the inlet of the SOURCE layer to initiate the assay. The horizontal diffusion in the SOURCE layer was estimated to render an input concentration ranging from 5 × 10^−3^ to 0.2 nM for different cylinder hydrogels after 12 h diffusion. After 2 days in the 3D culture, the hippocampal neurons showed little downward migration and developed significantly longer neurites, which were well aligned with the direction of increasing netrin-1 concentration. However, a similar growth pattern was observed over a large range of the netrin-1 gradient steepnesses across all the hydrogel cylinders (Fig. 3b–d). It is possible that the highly sensitive detection of netrin-1 molecules by neuronal growth cones^29^ and the spatial limit of the hydrogel cylinders could overwhelm the steepness-dependent growth under netrin-1 chemotactic guidance. We then performed the experiment of a shorter culture period (6 h), and found that the neurite length indeed showed some association with the steepness of netrin-1 gradient (*R*^2^ = 0.47, *p* = 1.21 × 10^−3^, *F*-test), but neuron migration and neurite steering were still steepness independent (Supplementary Fig. 3), suggesting that gradient steepness only plays minor role in netrin-1-regulated neuronal chemotaxis. In the statistical analysis, we fit a linear model to test the relationship between neuronal growth parameters and gradient steepness variation (slope-based measure, log-transformed). For NGF, the same experimental configuration was estimated to create an input concentration ranging from 0.04 to 0.4 nM for different cylinder hydrogels. We found that neuronal response to NGF gradient also showed more gradient steepness dependency, especially for neuronal migration (Fig. 3e–g). In hydrogel cylinders containing gradients with a steepness above 6.5% (~1.0 × 10^−3^ μm^−1^ in slope), the migration of neuron somata showed association with the gradient steepness (*R*^2^ = 0.63*, p* = 2.86 × 10^−5^, *F*-test), though such a tendency was not as obvious in moderate gradient steepness (Fig. 3f). The attractive axonal guidance effects from NGF appeared to be steepness-independent for the tested steepness and concentration range (Fig. 3g); significant attractive guidance was still observed for neurites in cylinders containing an NGF gradient as smooth as 0.3 × 10^−3^ μm^−1^ (*p* < 0.05, paired Kruskal–Wallis test). However, it is still possible that neurite growth cones can be sensitive to recognize small variations of NGF molecules under different background level of NGF^13,30^. In a control experiment where the guidance cues were presented by a uniform treatment (no gradient), neurons showed minimum chemotactic response in their migration, or guided neurite extension (Supplementary Fig. 4). Collectively, these demonstrations suggested that different growth parameters can be efficiently evaluated using our high-throughput chemotactic assays; our results also indicated a dramatic complexity for the involvement of gradient steepness in different aspects of neuronal chemotaxis.

### Steepness-dependent response to Sema3A

**Fig. 4:**
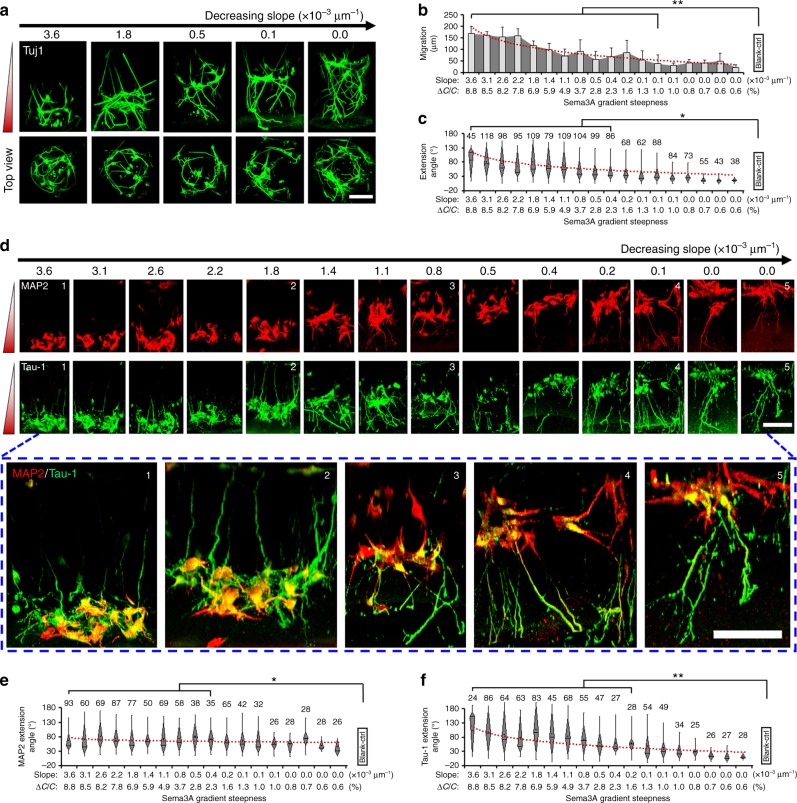
Neuronal response to Sema3A gradients with varied steepness. **a** Side and top view of 3D cultured neurons (β-tubulin) in response to Sema3A gradient of varied steepness, scale bar, 100 µm. **b** Quantitative analysis of neuronal migration and related association with the Sema3A gradient steepness, *n* = 6, error bars indicate standard deviation. **c** Box-plots for quantitative analysis of neurite repellence in response to varied Sema3A gradient steepness. More than 40 neurites (as indicated on top of each box) were pooled from four biological replicates. **d** Representative views of axonal (Tau-1^+^) or dendritic (MAP2^+^) differentiation under Sema3A gradient of decreasing steepness. The combined images of the indexed subpanels were enlarged and shown in the boxed region. Scale bar, 100 µm. **e**, **f** Box-plots for quantitative analysis of dendritic (**e**) or axonal (**f**) guidance in response to varied Sema3A gradient steepness. More than 20 neurites (as indicated on top of each box) were pooled from four biological replicates. For **c**, **e**, **f**, the parts of the box indicate 25, 50 and 75 percentiles, and the whiskers indicate 5% and 95%. The square mark indicates mean of the data. For **b**, **c**, **e** and **f**, the red line indicates a logarithmic fitting of the data mean; neuronal growth pattern in each hydrogel cylinder was compared in pairwise to experiments without any chemotactic factor treatment (Blank-ctrl, Supplementary Fig. 6), * or ** indicates a *p*-value < 0.05 or <0.005 by paired Kruskal–Wallis tests

After an initial validation with the above chemoattractive cues, we focused on a chemorepellent molecule, Sema3A, and used the HT-ChemoChip to dissect the role of its gradient steepness in regulating different but coordinated cellular programs (neuronal migration and neurite guidance). Estimated from our simulation results, the horizontal diffusion of Sema3A in the SOURCE layer was estimated to give an input concentration ranging from 1 × 10^−3^ to 0.05 nM for different cylinder hydrogels after 12 h diffusion, effectively built up Sema3A gradient with a steepness ranging from 1 × 10^-5^ to 3.6 × 10^-3^ μm^−1^ (or 0.6–8.8%). As shown in Fig. 4a, Sema3A exhibited more complex regulations on different aspects of neuronal development. The gradient of Sema3A molecules simultaneously repelled axonal growth (*R*^2^ = 0.80, *p* = 6.58 × 10^−7^, *F*-test) and promoted cellular migration (*R*^2^ = 0.95, *p* = 1.11 × 10^−13^, *F*-test) towards the higher concentration end in a steepness-dependent mode (Fig. 4b, c), which was not observed in different control groups (Supplementary Fig. 5). Under relatively steep gradients, neurons migrated to the bottom of the cylinder with their axons projected in the opposite direction towards the lower concentration end. The promotion of neuron migration was also accompanied by a neuron morphology transition from a multipolar to bipolar status (Fig. 4a, d), which consistent with a previous study that reported a coordinated program for migration and axonal projection in some pyramidal neurons^15^ and suggested multiple roles of Sema3A-associated chemotaxis in neural development^20,31^.

To analyze the level of neuronal differentiation and its relationship to Sema3A gradient steepness, we further performed immunostaining for axonal and dendritic markers (Tau-1 and MAP2) in neurons that were cultured in different Sema3A gradient. For hippocampal neurons cultured in traditional 2D substrates, axonal specification typically starts to be observable at 48 h after seeding^32^. Similarly, in our high-throughput 3D assays, the neurons showed some level of axonal specification and little dendritic growth after 2 days in vitro (DIV). Interestingly, we found that most of the neurites being strongly repelled by steep Sema3A gradient were the presumable axonal neurites (Tau-1^+^/MAP2^−^); some were even forced to grow upward against the gradient direction (Fig. 4d). Statistically, the axonal repellence was significantly associated with Sema3A gradient steepness (*R*^2^ = 0.95, *p* = 3.45 × 10^−12^, *F*-test), and was not observed for the presumed dendritic neurites (Tau-1^−^/MAP2^+^, Fig. 4e, f), though we cannot completely rule out the possibility that our experimental schedule (2 DIV) may be too early to detect any dendrite-associated regulations. Compared to the control (Blank-control, samples without any factors, Supplementary Fig. 6) or to the netrin-1 group (Supplementary Fig. 7), the growth of axonal neurites was substantially different, especially under steep gradient profiles. Altogether, these results well demonstrated that the steepness is a critical parameter for neuronal chemosensation to Sema3A gradient for different cellular programs in neuronal development, and also exemplify the advantages of our high-throughput chemotactic assay system for interrogating multiple coordinated cellular programs under a large-scale of different gradient profiles.

### Involvement of STK11 and GSK3 in sensing gradient steepness

Focusing on Sema3A-regulated neuron migration and neurite repellence, we then examined specific signaling pathways related to this cue and tried to dissect the signals that are responsible for sensing the Sema3A gradient steepness in different cellular programs. Particularly, there have been extensive but sometimes contradictory reports about the involvement of STK11 and GSK3 in the regulation of axon/dendrite outgrowth and cell migration^20,33–35^.

**Fig. 5:**
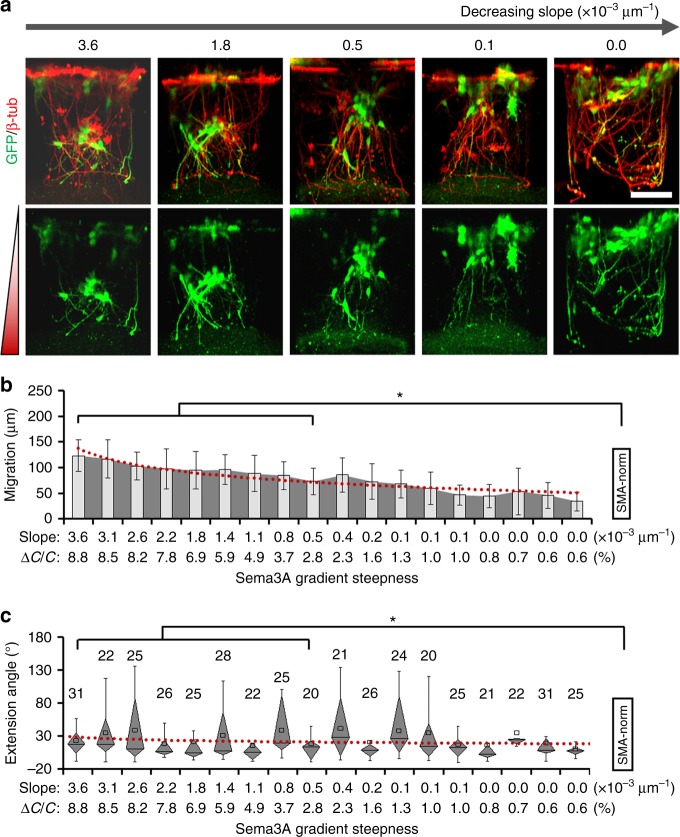
STK11 affects Sema3A-induced chemorepellence of neurite outgrowth. **a** Fluorescence images of neurons in a Sema3A gradient of various steepness after STK11 knockdown. Scale bar: 100 µm. **b** Quantitative analysis of STK11’s role in neuronal migration and related association with Sema3A gradient steepness, *n* = 3, error bars indicate standard deviation. **c** Box-plots for quantitative analysis of STK11’s role in the chemorepellent guidance of neurite outgrowth in response to varied Sema3A gradient steepness. The parts of the box indicate 25, 50, and 75 percentiles, and the whiskers indicate 5% and 95%. The square mark indicates mean of the data. More than 20 neurites (as indicated on top of each box) were pooled from three biological replicates. For **b**, **c**, the red line indicates logarithmic fitting of the data mean; neuronal growth pattern in each hydrogel cylinder was compared in pairwise to experiments with regular Sema3A treatment (SMA-norm), * indicates a *p*-value < 0.05 by paired Kruskal–Wallis tests

We first tested STK11’s role using the HT-ChemoChip. Small hairpin RNAs (shRNAs) were used to knockdown STK11 expression in the neurons that were cultured in the cylindrical hydrogel arrays (Fig. 5a, b), which significantly reduced Sema3A’s chemorepellent effect on neurite outgrowth, especially for large steepness profiles (Fig. 5c, *p* < 0.05, paired Kruskal–Wallis test). Most neurites were observed to grow downward in the same direction along the Sema3A gradient, probably resulting from the loss of axon formation after STK11 knockdown^35,36^. However, the same population of neurons (with STK11 knocked down) still showed strong migration towards the end of higher Sema3A concentration with a significant association with the steepness of the Sema3A gradient (Fig. 5b, *R*^2^ = 0.93, *p* = 1.38 × 10^−11^, *F*-test), though the overall migration was slightly reduced. These observations are in line with a previous report showing that conditional knockdown of STK11 leads to a loss in axon formation without affecting migration in cortical neurons^36^. Although we cannot draw a definitive conclusion about STK11’s role in the steepness-dependent regulation of axonal repellence, this experiment, at least, suggested that STK11 is not responsible for sensing the steepness variation of Sema3A gradient during neuronal migration.

**Fig. 6:**
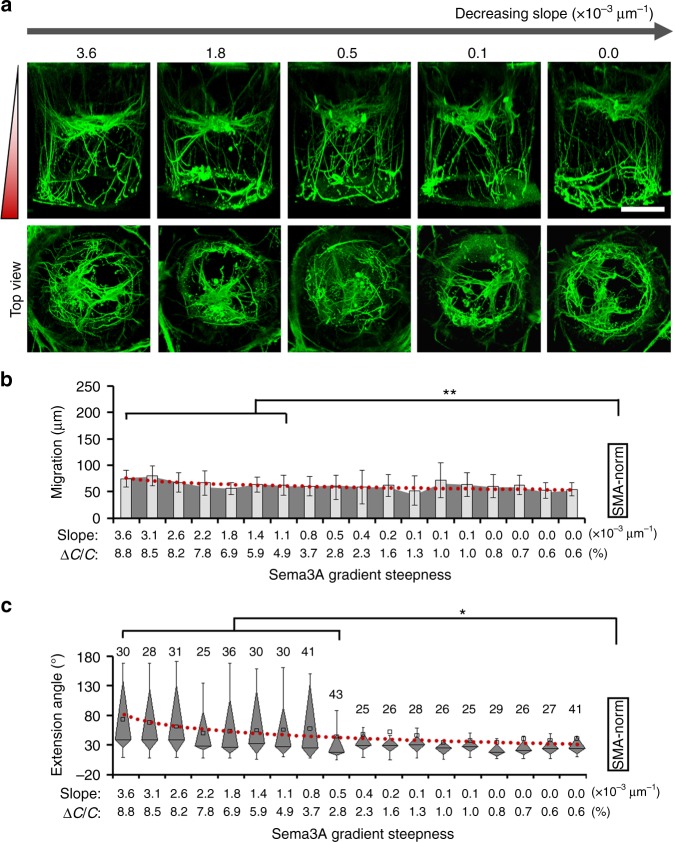
GSK3’s involvement in steepness-dependent regulation of neuronal migration. **a** Fluorescence images of neurons in Sema3A gradient of varied steepness after inhibition of the GSK3 activity. Scale bar: 100 µm. **b** Quantitative analysis of GSK3’s role in neuronal migration and related association with the Sema3A gradient steepness, *n* = 3, error bars indicate standard deviation. **c** Box-plots for quantitative analysis of GSK3’s role in the chemorepellent guidance of neurite outgrowth in response to the varied Sema3A gradient steepness. The parts of the box indicate 25, 50, and 75 percentiles, and the whiskers indicate 5% and 95%. The square mark indicates mean of the data. More than 25 neurites (as indicated on top of each box) were pooled from three biological replicates. For **b**, **c**, the dot-lines (red) indicate logarithmic fitting of the data mean; neuronal growth pattern in each hydrogel cylinder was compared in pairwise to experiments with regular Sema3A treatment (SMA-norm), * or ** indicates a *p*-value < 0.05 or <0.005 by paired Kruskal–Wallis tests

**Fig. 7:**
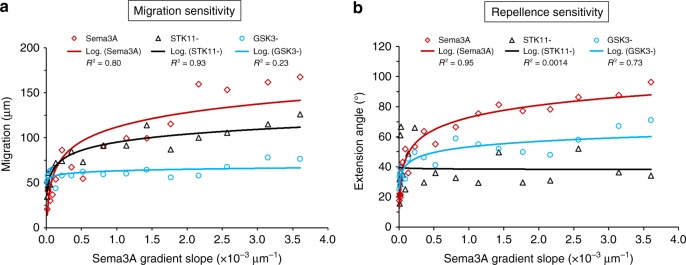
Summary of neuronal sensitivity to the steepness of Sema3A gradient. **a** Cell migration and **b** neurite repellence under different conditions, including Sema3A only (red, square), Sema3A plus STK11 knockdown (STK^−^, black, triangle), or Sema3A plus GSK3 inhibition (GSK3^−^, blue, circle). The solid lines indicate the logarithmic fitting of the data point. Data were quantified from at least three biological replicates. The association of neuronal growth (neurite guidance or soma migration) with the steepness of chemotactic gradient was examined using a linear fit for the neuronal growth parameters and gradient steepness variation. A *p*-value was calculated for the regression coefficient (gradient steepness), which, in a linear regression model, is based on a *t*-test with a null hypothesis that the coefficient is zero, and *p* < 1 × 10^−2^ indicates a significant association. The summary of *p*-values is included in Supplementary Table 1

We next examined GSK3, another kinase that has been reported to regulate the migration and morphology of cortical neurons^37^, possibly via semaphorin signaling pathways^4,38,39^. A small molecule, SB216763, was applied to inhibit GSK3 activity in the cultured hippocampal neurons. The inhibitor was added to the HT-ChemoChip via both the SOURCE and DRAIN layers to ensure a homogenous drug treatment to the neurons cultured in the hydrogel cylinders (Supplementary Fig. 8). We found that inhibition of GSK3 almost completely blocked migrating neurons’ sensitivity to Sema3A gradient steepness (Fig. 6a, b), even though the cells still showed more migration than Blank-control group without any chemotactic factors. However, the overall repellence of neurites from these neurons still showed association with the steepness of Sema3A gradient (Fig. 6c, *R*^2^ = 0.73, *p* = 1.39 × 10^−6^, *F*-test). These results directly indicated GSK3’s involvement in the steepness-dependent regulation of neuron migration by Sema3A (Fig. 7); and suggested a possible engagement of STK11 signaling for sensing the steepness variation of Sema3A gradient in the repellence of axonal outgrowth.

## Discussion

Here, we documented a systematic study of neuronal chemosensation to a large-scale variety of molecular gradient profiles and revealed an enormous complexity of neuronal chemotaxis, especially in response to the steepness variation of different chemotactic cues. This study was made feasible by an innovative design of a microfluidic device that is capable of generating large-scale molecular gradients with different levels of steepness in a microarray format, providing a high-throughput solution for investigating the dependence of neuronal chemotaxis on steepness of molecular gradient. In addition, the device utilizes arrays of hydrogel cylinders as the basic assay units, rendering a 3D ex vivo model to recapitulate the in vivo neuronal growth pattern.

From an instrumentation perspective, the device does not involve any complex fluidic handling components (e.g. valves and pumps) and is therefore very straightforward to operate^40–45^. The HT-ChemoChip only relies on a simple diffusion process to establish molecular gradients in a well-designed 3D space, in which the bottom horizontal gradient is used as varying inputs for each hydrogel cylinder to create gradients in the vertical direction to accommodate complex chemotactic assays. In addition, based on the working principle of the HT-ChemoChip, gradient profiles in a hydrogel can be further modulated by tuning different parameters governing the diffusion process, including diffusion coefficient, experimental temperature, medium viscosity, the background concentration level or the ending concentration for all cylinders (concentration in the DRAIN layer), and can potentially create a wide spectrum of different non-linear gradient profiles for different experimental requirements.

For the current study, the selection of steepness range for particular chemotactic molecules was mostly made by referencing to the literature. For example, NGF gradient steepnesses ranging from 0.1% to 0.4% has been reported to induce a differential response in DRG neurite outgrowth^13,30^. For Sema3A, though the gradient of endogenous Sema3A expression has previously been characterized in developing cortical tissues^4^, it is still difficult to have an exact quantification of the Sema3A concentration profile in a living mammalian brain. Another semaphorin protein, Sema2A, was documented to decrease by 2–4% of the maximum expression level over 10 μm (roughly 5–8% fractional change) in grasshopper’s tissue sections^46^. In cultured cortical slices of millimeter wide, the radial migration of progenitor cells can be significantly disrupted by a treatment of Sema3A at concentrations ranging from 0.1 to 1 μg/ml, which is achieved by masking the endogenous gradient expression of Sema3A^4^. Assuming that the exogenously applied Sema3A worked to block the lower or higher end of the endogenous Sema3A gradient with different concentrations, this experiment possibly suggests a difference of endogenous Sema3A expression in the range of 0–1 μg/ml along the radial cortical axis, which is roughly equivalent to a change of ~1% maximum expression level over 10 μm distance. With these considerations, the HT-ChemoChip was specifically designed to cover a wide relevant range of gradient steepnesses.

Recently, there is some skepticism regarding the extent to which axon guidance is regulated by molecular gradients in vivo, suggesting that chemotaxis is just one component of a set of mechanisms to produce appropriate brain wiring^47–49^. Indeed, using the high-throughput chemotactic assays, we showed that multiple levels of complexity exist in neuronal responses to chemotactic cues. First, neuronal chemotaxis exhibits a dramatic steepness-dependent variety in response to different guidance cues. For example, in addition to the extensively reported chemoattractive effects of netrin-1 and NGF on axonal growth^9,50^, NGF also showed significant regulation of neuronal migration in a steepness-dependent mode, which was not observed for netrin-1. Sema3A, on the other hand, exhibited steepness-dependent regulation for both neuronal migration and axonal repellence. Such a variety could potentially contribute to the combinatorial regulation of neuronal development via chemotaxis in a noisy extracellular environment^13,51^. Second, we found that neurite guidance and neuronal migration are closely related programs that can be simultaneously regulated by a single chemotactic molecule, but with different sensitivities to the gradient steepness. NGF can effectively promote neurite projection towards the higher concentration, even at a very low steepness, but it only enhances neuronal migration under relatively steep gradient conditions. Sema3A is a strong chemorepulsive cue for axonal growth, but works as a chemoattractant to promote neuronal migration over a large range of gradient steepness (~1% to 9% as quantified in fractional change). The coordination of the two cellular programs was also evidenced by a morphology change of neurons in Sema3A gradients with different steepnesses. A transition from multipolar to bipolar was observed as the steepness increased, which was accompanied by an increasing cellular migratory tendency. The multiple function of a single molecule adds additional complexity to understand neuronal chemotaxis.

Particularly for Sema3A, our observations of differential neuronal responses to various steepness profiles were confirmed and reinforced by a series of control experiments that ruled out possible false-positive observations. If the Sema3A proteins were heat-deactivated, the neurons almost completely lost their response to the molecules, despite the gradient profiles (Supplementary Fig. 5a–c). If a homogenous presentation of Sema3A was used in the HT-ChemoChip (no gradient, same concentration throughout cylinders across the whole chip), neurons only showed minimal migration and significantly less neurite repellence (Supplementary Fig. 5g–i), suggesting that the physical presentation of Sema3A in gradient is critical for its proper function^4,52^. If the Sema3A molecules was uniformly presented in the SOURCE layer (not in the DRAIN layer), and subsequently rendered a single gradient profile for all hydrogel cylinders across the whole chip, neuronal migration and neurite repellence were observed to be similar for all hydrogel cylinders (Supplementary Fig. 5d–f), supporting our conclusion of a gradient-steepness-dependent working principle for Sema3A.

In the second stage of this study, we focused on Sema3A and found that down-regulation of STK11 almost completely inhibited the steepness-dependence in neurite repellence, suggesting a potential role of STK11 in steepness sensation (Fig. 7). However, a similar phenotype can also result from a loss of axon formation due to STK11 knockdown^35,36^. For migration, we further showed that GSK3 is critical for neurons to recognize the Sema3A gradient steepness. Although inhibition of GSK3 or STK11 activity both reduces neuronal migration, a loss of sensitivity to gradient steepness was only observed in GSK3-inhibited neurons (Fig. 7a), which is consistent with recent literature reporting the essential role of GSK3 signaling in radial migration^37^. Collectively, these results suggest that different signaling pathways can be responsible for the steepness-dependent regulation of neurite guidance and neuronal migration; however, the results do not rule out the possibility that both cellular programs utilize a common upstream mechanism for sensing molecular steepness.

While this study is oriented to dissect the role of Sema3A gradient steepness, our results do not exclude more complexity of Sema3A-regulated neuronal chemotaxis that involves contribution from both absolution concentration and gradient steepness of a particular chemotactic molecule, as demonstrated by Mortimer et al.^30^ in their theoretical and experimental investigations. For different gradient steepness, they nicely showed that neurite guidance depends on the concentration of NGF following a Bayesian model^30^. Actually, molecular concentration, gradient steepness, and neuronal responsive range are inter-correlated elements, and it is easier to dissociate gradient steepness and absolute concentration variables under relatively shallow gradients^13,30^. For this study, we focused on dissecting the role of gradient steepness (especially for Sema3A) in regulating different but coordinated cellular programs (neuronal migration and neurite guidance), and therefore seeded the neurons on the top of the hydrogel cylinders, which effectively renders a similar starting concentration (close to zero) of the chemotactic factors for cells in different hydrogel cylinders. Certainly, using the same setup, the concentration-related regulation could be further explored in more details by adjusting the background concentration level of a guidance factor.

In sum, we developed a microfluidic platform that enables high-throughput 3D chemotactic assays, which we used to systematically study neurons’ sensitivity to the steepness of different guidance molecules and revealed dramatic diversity and complexity in relevant regulation of neuronal migration and axonal projection. These results provide insights regarding the role of gradient steepness in neuronal chemotaxis. We believe that the 3D high-throughput chemotactic assay platform provides an innovative experimental framework to advance the field of neurobiology.

## Methods

### Fabrication of microfluidic device

The microfluidic device was made by assembling a polydimethylsiloxane (PDMS) chamber (the DRAIN layer), PDMS stencil (STENCIL layer), PDMS membrane (SOURCE layer), and standard glass slide. The PDMS stencil was replica molded from a master, which consisted of one micropatterned SU-8 (Microchem) layer on a silicon wafer^24^. The SU-8 layer contained an array of cylinders that each measured 250 µm in height and 200 µm in diameter. To create the through structures in the stencil, a thin layer of silane-treated (trichloro-perfluorooctyl silane, Sigma) PDMS blanket was placed on the SU-8 features to prevent the top from contacting prepolymer of PDMS, which was then poured onto the mold and degassed. Then, a plastic transparency was carefully lowered onto the prepolymer. The stack containing SU-8 mold, protection blanket, PDMS prepolymer, and transparency was clamped within two flat aluminum plates, and was baked at 80 °C for 12 h. At disassembly, the transparency and the protection blanket were carefully removed before releasing the stencil (10–60 µm thick) from the mold. The PDMS membrane was fabricated by replica molding of a CNC machined copper mold. The working principle and assembly process is illustrated in Fig. 1. The PDMS stencil was first glued to the bottom of the PDMS chamber and then gently placed against a clean glass slide. The whole unit was plasma treated for 1 min to render the top surface and the through-holes hydrophilic while keeping the bottom surface hydrophobic. Next, 10 μl of a Matrigel (Invitrogen) aqueous solution was pipetted onto the bottom side (hydrophobic) of the PDMS stencil and was evenly spread across the surface. Each through-hole was filled with the Matrigel aqueous solution due to capillary force. Upon further incubation at 37 °C, the Matrigel solution in the through-holes solidified and formed hydrogel cylinders. The residual Matrigel on the bottom surface was then removed. To assemble the device, the PDMS membrane (SOURCE layer) was sandwiched between a glass slide and the top unit (PDMS chamber bottom sealed by the PDMS stencil filled with Matrigel), forming the final device for the high-throughput chemotactic assay.

### Computational simulation of gradient generation

Simulation of gradient generation was performed in Matlab using our custom-developed code. Specifically, diffusion in the 3D microfluidic device was separated into two connected systems, which were analyzed by two one-dimensional models. In the bottom SOURCE layer, the horizontal spreading of molecules from the inlet follows the normal diffusion equation, assuming a constant diffusion coefficient, *D*_0_ = 57 μm^2^/s, in aqueous culture medium^53^. The hydrogel we used in this study was the stock solution of Geltrex^TM^ derived from basement membrane matrix with a total protein concentration of 15.6 mg/ml. This highly concentrated solute proteins effectively work as obstacles and provide a crowded environment for a chemotactic protein to diffuse. Therefore, in the hydrogel cylinders, the vertical anomalous diffusion of molecules was simulated using in a stretched exponential model featuring a concentration-dependent diffusion coefficient^54^, which was derived from the experimental data. More details are included in the Supplementary Note 1.

### Experimental characterization of gradient generation

The generation of the molecular gradient was characterized by fluorescence microscopy. After the addition of 70-kDa FITC-dextran (Sigma) to the inlet of the channel, the device was put into a wet Petri dish and brought to the incubator to prevent evaporation, and fluorescent images were taken by a confocal scanning laser microscope (TCS SP8, Leica Microsystems) at 7, 12, and 24 h time points. By fixing the laser power and scanning speed, the standard curve to describe the relationship between dextran concentrations and fluorescence intensities was generated at the same time. Image processing of the fluorescent images was performed using ImageJ software to obtain the changes in fluorescent intensity across the gel at each time point. The dextran concentrations were calculated based on the standard curve.

### Cell culture

Hippocampal neuron cultures were used in this study. Dissociated neurons were prepared from hippocampi dissected from E18 Sprague Dawley rats. All animal procedures were approved by the Animal Ethics Committee of the City University of Hong Kong. The isolated tissues were treated with papain (Sigma) for 30 min at 37 °C followed by trituration with a 1 ml pipette tip. The cell solution was then extracted. For each device, 80,000 neurons were added to the DRAIN layer and seeded onto the top surface of the suspended STENCIL layer. The culture was maintained in Neurobasal medium (Invitrogen) supplemented with B27, l-glutamine and penicillin/streptomycin. 24 h after cell seeding, different guidance molecules, netrin-1 (5 ng), NGF (1 ng), and Sema3A (5 ng), were introduced to the device via the inlet of the SOURCE layer to initiate the chemotactic assays. The growth of neurons was then examined 24 h later. All of these guidance molecules were acquired from R&D systems.

### Immunocytochemistry

Before microscopic examination, the neuron cultures (in the microfluidic device) were fixed for 30 min in 4% paraformaldehyde in phosphate-buffered saline (PBS), permeabilized in 0.25% Triton X-100 for 30 min and then blocked with 4% bovine serum albumin (BSA) in PBS for 1 h at room temperature. The cultures were incubated with primary antibodies (diluted in 1% casein) for 4 h at room temperature and then with secondary antibodies for 2 h. All excessive antibodies were removed by rinsing with PBS after each incubation period. The primary antibodies included mouse anti-βIII tubulin (R&D, MAB1195; 1:500), rabbit anti-GFP (Thermo Fisher, A21311; 1:500), chicken anti-MAP2 (Abcam, AB5392; 1:2000), and mouse anti-Tau-1 (Millipore, MAB3420; 1:500).

### Signaling pathway analysis

To knock down STK11 expression in the cultured neurons, sh-LTviral-STK11 lentivirus particles (ATCGbio) were added to the culture 24 h after cell seeding and incubated with the cells for 12 h before introduction of Sema3A. The virus concentration is ~0.18 × 10^8^ TU/ml. To inhibit GSK3 activity in the cultured neurons, the inhibitor (SB216763) was added to the culture at 5 μM 24 h after cell seeding and maintained for 24 h throughout the assay.

### Confocal microscopy and quantification of neuron growth

After immunostaining, the samples were imaged using a confocal laser scanning microscope (TCS SP8, Leica Microsystems) equipped with a ×40 water immersion objective. For quantitative analysis, all of the imaging parameters, including the laser power, gain, and scanning speed were fixed across the microscopy process. For each hydrogel cylinder, scanning was performed with a 1 μm step size along the *Z*-axis spanning the whole hydrogel height. 3D reconstruction was then performed to visualize the growth of neuron cells in the hydrogel cylinders. The images were quantitatively analyzed using Image J software.

To quantify the neurite extension angle, a *YZ*-projection (side view) image was generated from the 3D reconstruction of a confocal scan. All visibly differentiable neurites are measured for analysis. The vertical downward direction of *Z*-axis was used as a reference. For each neurite, an auxiliary line was firstly drawn between the end of the neurite and its starting point on the neuron soma; the angle between the auxiliary line and the reference axis was used as an indicator of the neurite guidance level. For a chemoattractive factor, the neurites extension angle should be small and close to zero. For a chemorepellent factor, the extension angle should increase accordingly. In some cases with a steep Sema3A gradient, the neurites were repelled to grow upward (opposite to the gradient direction), their extension angle could be larger than 90° (Fig. 1).

### Statistical analysis

At each hydrogel location (ranging from 0.3 to 7.9 mm away from the inlet spaced by 0.4 mm), pairwise Kruskal–Wallis test was performed at each hydrogel cylinder location to determine the statistical significance between the experimental conditions and the control groups, *p* < 0.05 indicates a significant difference. For quantitative analysis, at least three biological replicates were used in this study. From each replicate, 2–3 rows of hydrogel cylinders were quantified; in each hydrogel, 5–10 neurons were measured to access the neurite growth and migration level. For Fig. 3, the condition with netrin-1 or NGF gradient was compared to samples without any factor treatment (Blank-control). For Fig. 4, the condition with Sema3A gradient was compared to Blank-control. For Figs. 5 and 6 the experimental condition (Sema3A gradient and STK11 knockdown, Sema3A gradient and GSK inhibitor) was compared to samples under regular Sema3A gradient (SMA-Norm). In our study, the dependence of neuronal growth (neurite length, guidance, or migration) on the steepness of chemotactic gradient was examined by fitting a linear model for the neuronal growth parameters (e.g. length, guidance, and migration) and gradient steepness variation (slope-based measure, log-transformed). The reported *p*-value was calculated for the regression coefficient of the logarithm of gradient steepness, which, in a linear regression model, is based on a *t*-test with a null hypothesis that the coefficient is zero, and *p* < 0.01 indicates a significant association. *R*^2^ along with the *p*-value were derived from *F*-test for the overall statistical significance of the linear fitting (Supplementary Table 1).

## Electronic supplementary material

Supplementary Information

Description of Additional Supplementary Files

Supplementary Movie 1

Source Data

Reporting Summary

## Data Availability

The data that support the findings of this study are available from the corresponding author on request. The source data underlying Fig. 2a–c are provided as a Source Data file.

## References

[1] Tessier-Lavigne M, Goodman CS (1996). The molecular biology of axon guidance. Science.

[2] Chedotal A, Richards LJ (2010). Wiring the brain: the biology of neuronal guidance. Cold Spring Harb. Perspect. Biol..

[3] Marin O, Valiente M, Ge X, Tsai LH (2010). Guiding neuronal cell migrations. Cold Spring Harb. Perspect. Biol..

[4] Chen G (2008). Semaphorin-3A guides radial migration of cortical neurons during development. Nat. Neurosci..

[5] Ming GL (1997). cAMP-dependent growth cone guidance by netrin-1. Neuron.

[6] Tamagnone L, Comoglio PM (2000). Signalling by semaphorin receptors: cell guidance and beyond. Trends Cell Biol..

[7] Brose K, Tessier-Lavigne M (2000). Slit proteins: key regulators of axon guidance, axonal branching, and cell migration. Curr. Opin. Neurobiol..

[8] Guan KL, Rao Y (2003). Signalling mechanisms mediating neuronal responses to guidance cues. Nat. Rev. Neurosci..

[9] Gundersen RW, Barrett JN (1979). Neuronal chemotaxis: chick dorsal-root axons turn toward high concentrations of nerve growth factor. Science.

[10] Dupin I, Dahan M, Studer V (2013). Investigating axonal guidance with microdevice-based approaches. J. Neurosci..

[11] Nedelec S (2012). Concentration-dependent requirement for local protein synthesis in motor neuron subtype-specific response to axon guidance cues. J. Neurosci..

[12] Manns RP, Cook GM, Holt CE, Keynes RJ (2012). Differing semaphorin 3A concentrations trigger distinct signaling mechanisms in growth cone collapse. J. Neurosci..

[13] Rosoff WJ (2004). A new chemotaxis assay shows the extreme sensitivity of axons to molecular gradients. Nat. Neurosci..

[14] Mortimer D (2010). Axon guidance by growth-rate modulation. Proc. Natl Acad. Sci. USA.

[15] Noctor SC, V MC, Ivic L, Kriegstein AR (2004). Cortical neurons arise in symmetric and asymmetric division zones and migrate through specific phases. Nat. Neurosci..

[16] Song H, Poo M (2001). The cell biology of neuronal navigation. Nat. Cell Biol..

[17] Xu Z (2018). Regeneration of cortical tissue from brain injury by implantation of defined molecular gradient of semaphorin 3A. Biomaterials.

[18] Zheng W, Geng AQ, Li PF, Wang Y, Yuan XB (2012). Robo4 regulates the radial migration of newborn neurons in developing neocortex. Cereb. Cortex.

[19] Serafini T (1996). Netrin-1 is required for commissural axon guidance in the developing vertebrate nervous system. Cell.

[20] Shelly M (2011). Semaphorin3A regulates neuronal polarization by suppressing axon formation and promoting dendrite growth. Neuron.

[21] Pujic Z, Giacomantonio CE, Unni D, Rosoff WJ, Goodhill GJ (2008). Analysis of the growth cone turning assay for studying axon guidance. J. Neurosci. Methods.

[22] Morel M (2012). Amplification and temporal filtering during gradient sensing by nerve growth cones probed with a microfluidic assay. Biophys. J..

[23] Ming GL (2002). Adaptation in the chemotactic guidance of nerve growth cones. Nature.

[24] Li W (2014). NeuroArray: a universal interface for patterning and interrogating neural circuitry with single cell resolution. Sci. Rep..

[25] Li Wei, Xu Zhen, Xu Bingzhe, Chan Chung Yuen, Lin Xudong, Wang Ying, Chen Ganchao, Wang Zhigang, Yuan Qiuju, Zhu Guangyu, Sun Hongyan, Wu Wutian, Shi Peng (2017). Investigation of the Subcellular Neurotoxicity of Amyloid-β Using a Device Integrating Microfluidic Perfusion and Chemotactic Guidance. Advanced Healthcare Materials.

[26] Shi P (2011). Synapse microarray identification of small molecules that enhance synaptogenesis. Nat. Commun..

[27] Wang Y (2014). Poking cells for efficient vector-free intracellular delivery. Nat. Commun..

[28] Dickson BJ (2002). Molecular mechanisms of axon guidance. Science.

[29] Pinato G (2012). Less than 5 Netrin-1 molecules initiate attraction but 200 Sema3A molecules are necessary for repulsion. Sci. Rep..

[30] Mortimer D (2009). Bayesian model predicts the response of axons to molecular gradients. Proc. Natl Acad. Sci. USA.

[31] Cheadle L, Biederer T (2014). Activity-dependent regulation of dendritic complexity by semaphorin 3A through Farp1. J. Neurosci..

[32] Kaech S, Banker G (2006). Culturing hippocampal neurons. Nat. Protoc..

[33] Asada N, Sanada K, Fukada Y (2007). LKB1 regulates neuronal migration and neuronal differentiation in the developing neocortex through centrosomal positioning. J. Neurosci..

[34] Asada N, Sanada K (2010). LKB1-mediated spatial control of GSK3beta and adenomatous polyposis coli contributes to centrosomal forward movement and neuronal migration in the developing neocortex. J. Neurosci..

[35] Shelly M, Cancedda L, Heilshorn S, Sumbre G, Poo MM (2007). LKB1/STRAD promotes axon initiation during neuronal polarization. Cell.

[36] Barnes AP (2007). LKB1 and SAD kinases define a pathway required for the polarization of cortical neurons. Cell.

[37] Morgan-Smith M, Wu Y, Zhu X, Pringle J, Snider WD (2014). GSK-3 signaling in developing cortical neurons is essential for radial migration and dendritic orientation. eLife.

[38] Renaud J (2008). Plexin-A2 and its ligand, Sema6A, control nucleus-centrosome coupling in migrating granule cells. Nat. Neurosci..

[39] Nakamura F (2009). Increased proximal bifurcation of CA1 pyramidal apical dendrites in sema3A mutant mice. J. Comp. Neurol..

[40] Beck C, Singh T, Farooqi A, Venkatesh T, Vazquez M (2016). Controlled microfluidics to examine growth-factor induced migration of neural progenitors in the *Drosophila* visual system. J. Neurosci. Methods.

[41] Keenan TM, Folch A (2008). Biomolecular gradients in cell culture systems. Lab Chip.

[42] Bhattacharjee N, Folch A (2017). Large-scale microfluidic gradient arrays reveal axon guidance behaviors in hippocampal neurons. Microsyst. Nanoeng..

[43] Sackmann EK, Fulton AL, Beebe DJ (2014). The present and future role of microfluidics in biomedical research. Nature.

[44] Abhyankar VV (2008). A platform for assessing chemotactic migration within a spatiotemporally defined 3D microenvironment. Lab Chip.

[45] Jeon NL (2002). Neutrophil chemotaxis in linear and complex gradients of interleukin-8 formed in a microfabricated device. Nat. Biotechnol..

[46] Isbister CM, Mackenzie PJ, To KC, O’Connor TP (2003). Gradient steepness influences the pathfinding decisions of neuronal growth cones in vivo. J. Neurosci..

[47] Goodhill GJ (2016). Can molecular gradients wire the brain?. Trends Neurosci..

[48] Dominici C (2017). Floor-plate-derived netrin-1 is dispensable for commissural axon guidance. Nature.

[49] Varadarajan SG (2017). Netrin1 produced by neural progenitors, not floor plate cells, is required for axon guidance in the spinal cord. Neuron.

[50] Graef IA (2003). Neurotrophins and netrins require calcineurin/NFAT signaling to stimulate outgrowth of embryonic axons. Cell.

[51] Bicknell BA, Dayan P, Goodhill GJ (2015). The limits of chemosensation vary across dimensions. Nat. Commun..

[52] Polleux F, Morrow T, Ghosh A (2000). Semaphorin 3A is a chemoattractant for cortical apical dendrites. Nature.

[53] Braga J, Desterro JM, Carmo-Fonseca M (2004). Intracellular macromolecular mobility measured by fluorescence recovery after photobleaching with confocal laser scanning microscopes. Mol. Biol. Cell.

[54] Banks DS, Fradin C (2005). Anomalous diffusion of proteins due to molecular crowding. Biophys. J..

