# Supplementary material for: High-throughput three-dimensional chemotactic assays reveal steepness-dependent complexity in neuronal sensation to molecular gradients
Source: Nat Commun. 2018 Nov 12;9:4745. doi: 10.1038/s41467-018-07186-x (PMC6232128; doi:10.1038/s41467-018-07186-x)
Supplement: Supplementary file 1 — Supplementary Information [file 41467_2018_7186_MOESM1_ESM.pdf]

Supplementary Information

High-throughput Three-dimensional Chemotactic Assays Reveal Steepness-dependent Complexity in Neuronal Sensation to Molecular Gradients

Zhen Xu[1,¶], Peilin Fang[1,¶], Bingzhe Xu[1,¶], Yufeng Lu[2], Jinghui Xiong[2], Feng Gao[3], Xin Wang[3,4], Jun Fan[2], Peng Shi[1,4,*]

[1]Department of Biomedical Engineering
City University of Hong Kong,
Kowloon, Hong Kong SAR

[2]Department of Material Science and Engineering
City University of Hong Kong,
Kowloon, Hong Kong SAR

[3]Department of Biomedical Science
City University of Hong Kong,
Kowloon, Hong Kong SAR

[4]Shenzhen Research Institute
City University of Hong Kong,
Shenzhen, China, 518000

*Correspondence should be addressed to Prof. Peng Shi, pengshi@cityu.edu.hk

[¶]These authors contributed equally to this work.

Supplementary Note 1

Details of 3D-Diffusion modeling

In our simulation of the 3D diffusion process, we simplify the 3D diffusion into two 1D models with the following assumptions:

- There is no flux along Y-direction in the bottom horizontal plane (source layer).
- The concentration variation within the XY plane of a hydrogel cylinder was neglected, so that there is only flux along Z-direction in each cylinder.
- The cell seeding chamber (drain layer) was sufficiently large, so that the overall concentration in the chamber is not affected by the diffusion and remains close to zero over the experimental period.

Therefore, the diffusion in the bottom layer (source layer) is governed by the regular diffusion equation:

$$\frac{\partial c_{bottom}}{\partial t} = D_0 \frac{\partial^2 c_{bottom}}{\partial x^2} \qquad (1)$$

and the diffusion in the hydrogel cylinder is governed by the following equation:

$$\frac{\partial c_n}{\partial t} = \frac{\partial}{\partial z}\left(D_n \frac{\partial c_n}{\partial z}\right) \qquad (2)$$

where x represents the distance from the inlet of the device, z represents the distance from the bottoms of the hydrogel cylinder, D_0 denotes the diffusion coefficient of the molecules in low viscosity solutions (e.g. culture medium) and D_n denotes the diffusion coefficient of the same molecules in very viscous medium (e.g. hydrogel); n represent the number of the hydrogel cylinder.

The solution for equation (1) can be expressed as:

$$c_{bottom}(x,t) = \frac{C_0}{\sqrt{4\pi D_0 t}} exp\left(-\frac{x^2}{4D_0 t}\right) \qquad (3)$$

which describes the Gaussian distribution evolved from a delta function input (addition of molecules to the inlet). This solution also gives the initial starting molecule concentration of each cylinder at the interface between aqueous culture medium and viscous hydrogels. In our simulation, the diffusion coefficient of the 70 kDa dextran, D_o, was assumed to be 57 $\mu m^2/s$ by referencing to the literature [1, 2].

Notably, in the polymer-like materials, molecular diffusion is more complex, as D_n has been reported to depend on the local concentration c due to molecular crowding [3, 4, 5]. We employed an exponential model that has been proposed by Banks *et al* to describe such anomalous diffusion in concentrated hydrogels [5]:

$$D_n = D_0 exp(-\beta c^{\gamma}) \qquad (4)$$

where β and γ was determined to be *11.39* and *0.20* by fitting the experimental data.

Supplementary Figures

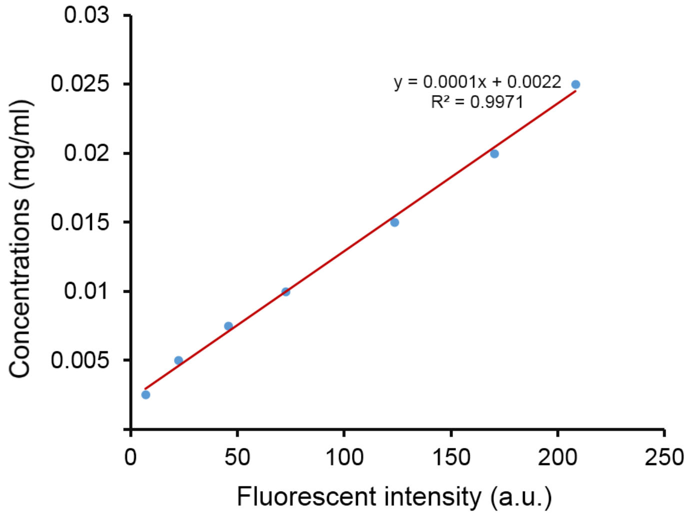

Supplementary Figure 1. A representative standard curve showing the linear correlation between the concentration between fluorescent labeled dextran (70KDa) and the acquired fluorescence intensity by confocal microscopy.

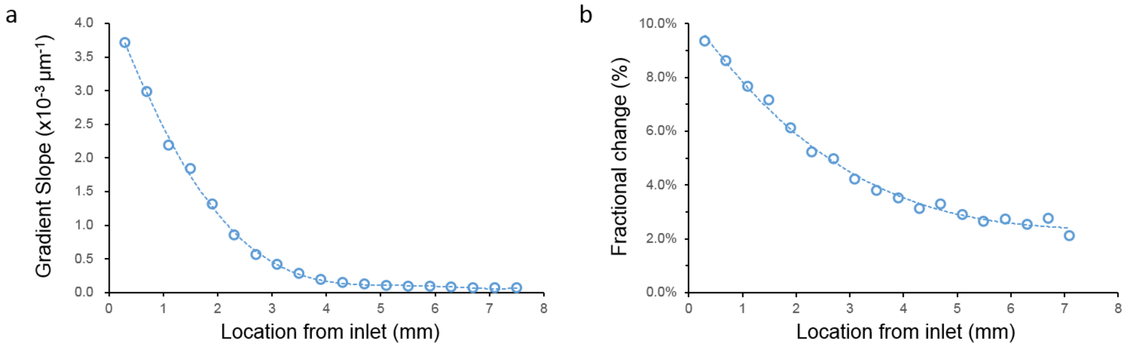

Supplementary Figure 2. The variation of steepness for molecular gradient established by the diffusion of 70 kDa dextran in hydrogel cylinders of 0.3 ~ 7.1 mm from the inlet of a HT-ChemoChip. The steepness is quantified by (**a**) slope of linear-fit or (**b**) fractional change over 10 μm derived from exponential-fit of the concentration curves.

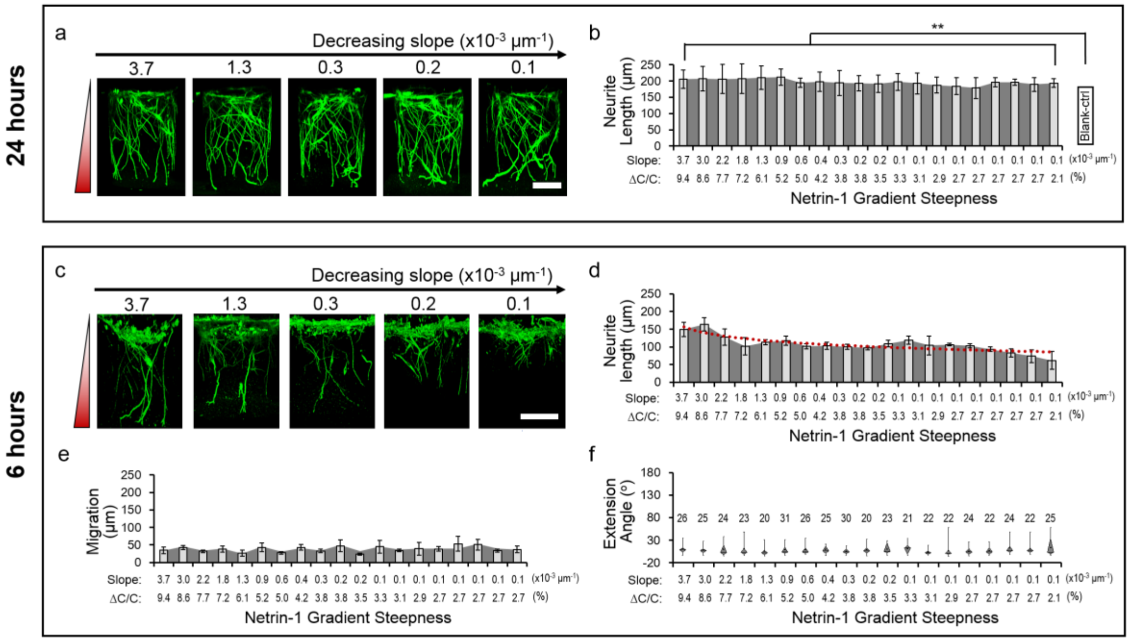

Supplementary Figure 3. Neurite outgrowth sensitive to netrin-1 gradient of varied steepness at 24 hours and 6 hours. (**a**) Side view of 3D cultured hippocampal neurons in response to netrin-1 gradient of different steepness at 24 hours, scale bar, 100 μm. (**b**) Quantitative analysis of neurite length and related dependence on netrin-1 gradient steepness at 24 hours, n = 4, error bars indicate the standard deviation (SD). The neuronal growth pattern in each hydrogel cylinder was compared in a pairwise manner to experiments without any chemotactic factor treatment (Blank-ctrl, Supplementary Fig. 6), ** indicates a *p*-value < 0.005 by paired Kruskal-Wallis test. (**c**) Side views of 3D cultured neurons in response to netrin-1 gradient of varied steepness at 6 hours. Scale bar, 100 μm. (**d**) Quantitative analysis of neurite length and related dependence on netrin-1 gradient steepness 6 hours after seeding, n = 3, error bars indicate SD. The red line indicates logarithmic fitting of the data mean, the dependence between neurite length and netrin-1 steepness is significant at 6 hours after seeding, $R^2 = 0.47$, $p = 1.2 \times 10^{-3}$, F-test. (**e**) Quantitative analysis of neuronal migration and related dependence on netrin-1 gradient steepness 6 hours after seeding, n = 3, error bars indicate SD. (**f**) Box-plots for quantitative analysis of neurite guidance in response to varied netrin-1 gradient steepness at 6 hours. The parts of the box indicate 25, 50 and 75 percentiles, and the whiskers indicate 10 and 90%. The square mark indicates mean of the data. About 20 neurites (as indicated on top of each box) were pooled form 3 biological replicates.

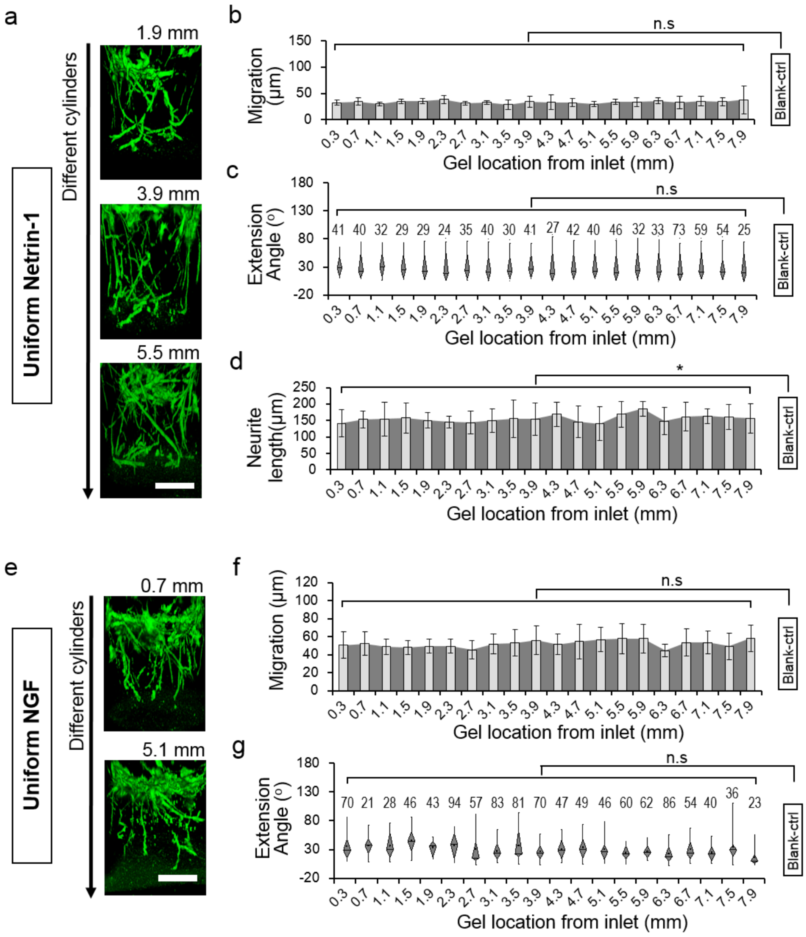

Supplementary Figure 4. Neuronal response to homogenous treatment of netrin-1 (**a-c,** 0.01 nM) or NGF (**d-f**, 0.05 nM). (**a, e**) Side view of cultured neurons (stained for β-tubulin) in response to homogenously presented netrin-1 (**a**) or NGF (**e**), scale bar, 100 μm. (**b, f**) Quantitative analysis of neuronal migration in response to homogenously presented netrin-1 (**b**) or NGF (**f**). (**c, g**) Box-plots for quantitative analysis of neurite guidance in response to homogenously presented netrin-1 (**c**) or NGF (**g**). The parts of the box indicate 25, 50 and 75 percentiles, and the whiskers indicate 10 and 90%. The square mark indicates data mean. More than 20 neurites (as indicated on top of each box) were pooled from three biological replicates. The neuronal growth pattern in each hydrogel cylinder was compared in pairwise to experiments without any chemotactic factor treatment (Blank-ctrl, Supplementary Fig. 6), * indicates $p < 0.05$ by paired Kruskal-Wallis test. (**d**) Quantitative analysis of neurite length in response to homogenously presented netrin-1. n = 3, error bars indicate standard deviation in panel **b**, **d** & **f**.

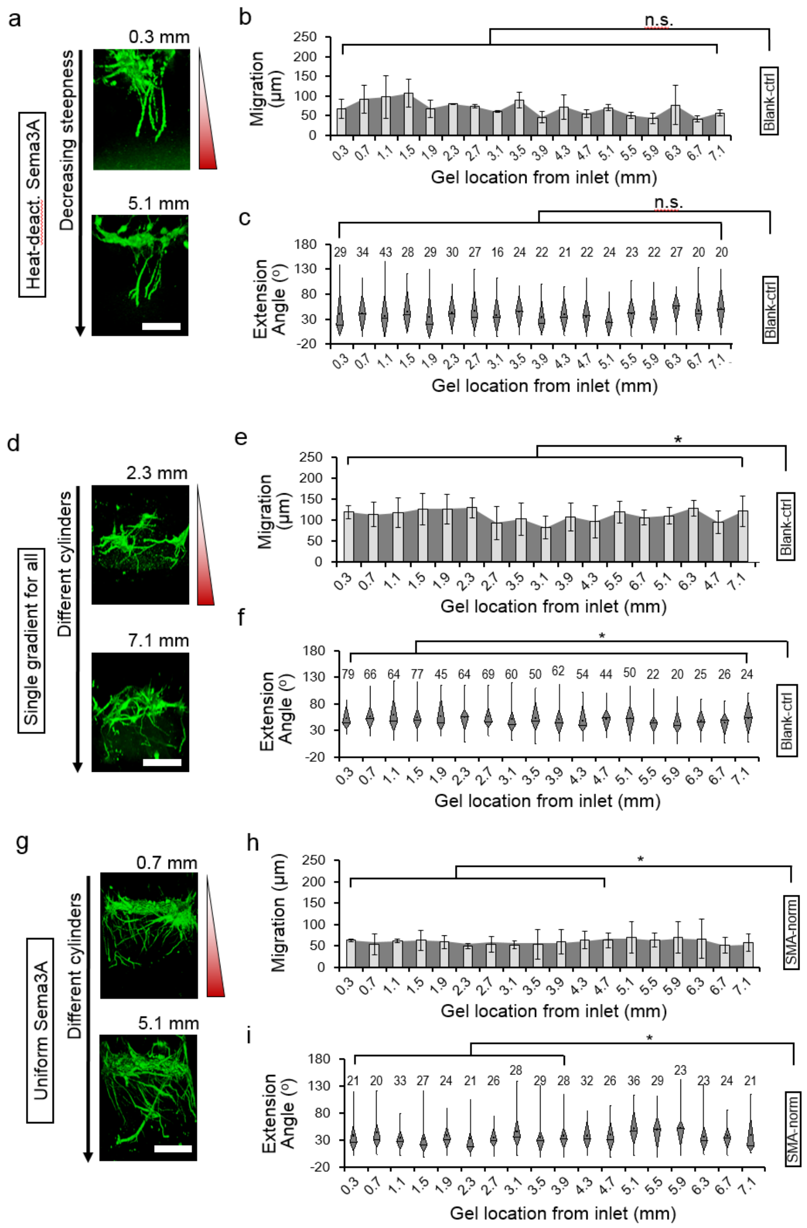

Supplementary Figure 5. Neuronal responses to different forms of Sema3A: (**a-c**) heat-deactivated Sema3A (5 ng to the inlet) gradients; (**d-e**) same single Sema3A gradient for all cylinders (2.5 ng/ml, 0.02 nM in the source layer only); (**g-i**) uniform treatment of Sema3A (no gradient) for all cylinders (2.5 ng/ml, 0.02 nM)**.** (**a, d, g**) Side view of cultured neurons stained for β-tubulin in different control conditions, scale bar, 100 µm. (**b, e, h**) Quantitative analysis of neuronal migration, n=3, error bars indicate standard deviation. (**c, f, i**) Box-plots for quantitative analysis of neurite guidance. The parts of the box indicate 25, 50 and 75 percentiles, and the whiskers indicate 10 and 90%. The square mark indicates data mean. More than 20 neurites (as indicated on top of each box) were pooled from three biological replicates. For heat-deactivated Sema3A and single gradient conditions, the neuronal growth pattern in each hydrogel cylinder was compared in pairwise to experiments without any chemotactic factor treatment (Blank-ctrl, Supplementary Fig. 6). For Uniform Sema3A condition, the neuronal growth pattern in each hydrogel cylinder was compared in pairwise to experiments with regular Sema3A gradient of varied steepness (SMA-norm). n.s. indicates no significance ($p > 0.05$) by paired Kruskal-Wallis test.

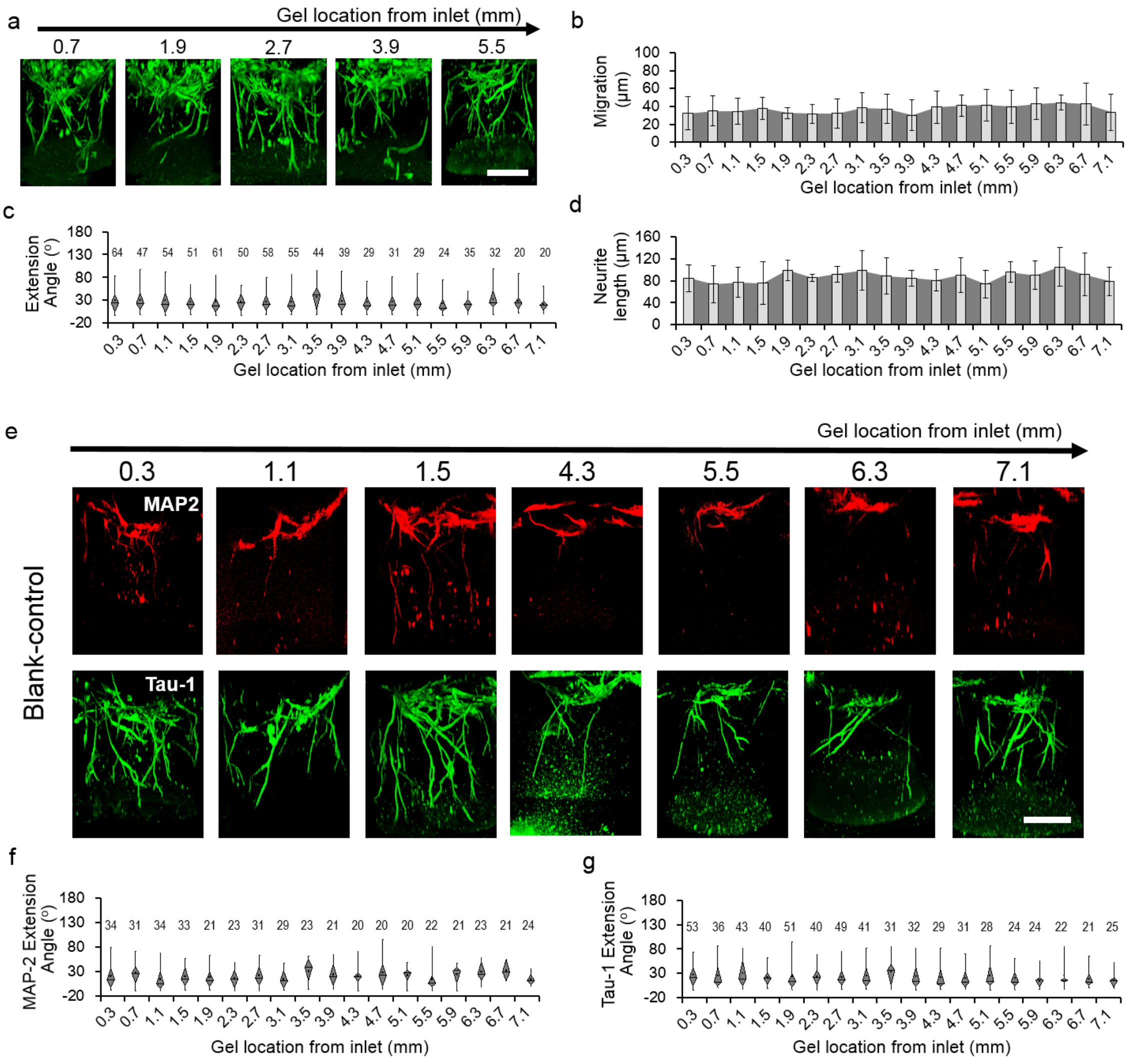

Supplementary Figure 6. Summary of neuronal growth in the HT-ChemoChip without any treatment of chemotactic factor at 24 hours (the Blank-control group). (**a**) Representative fluorescence images showing neurons stained for β-tubulin, scale bar, 100 μm. (**b**) Quantitative analysis of neuronal migration in the Blank group. (**c**) Box-plots for quantitative analysis of neurite outgrowth in the Blank group. (**d**) Quantitative analysis of neuronal length in the Blank group. (**e**) Representative side view of neurons cultured in hydrogel cylinders of different distance to the inlet; the neurons were stained for axonal (Tau-1) and dendritic markers (MAP2), scale bar, 100 μm. (**f, g**) Box-plots for quantitative analysis of dendritic (**f**) or axonal (**g**) guidance in response to varied Sema3A gradient steepness. For **a** & **c**, n = 4, error bars indicate SD. For **c, f, g**, more than 20 neurites (as indicated on top of each box) were pooled from 4 biological replicates; the parts of the box indicate 25, 50 and 75 percentiles, and the whiskers indicate 10 and 90%, and the square mark indicates mean of the data.

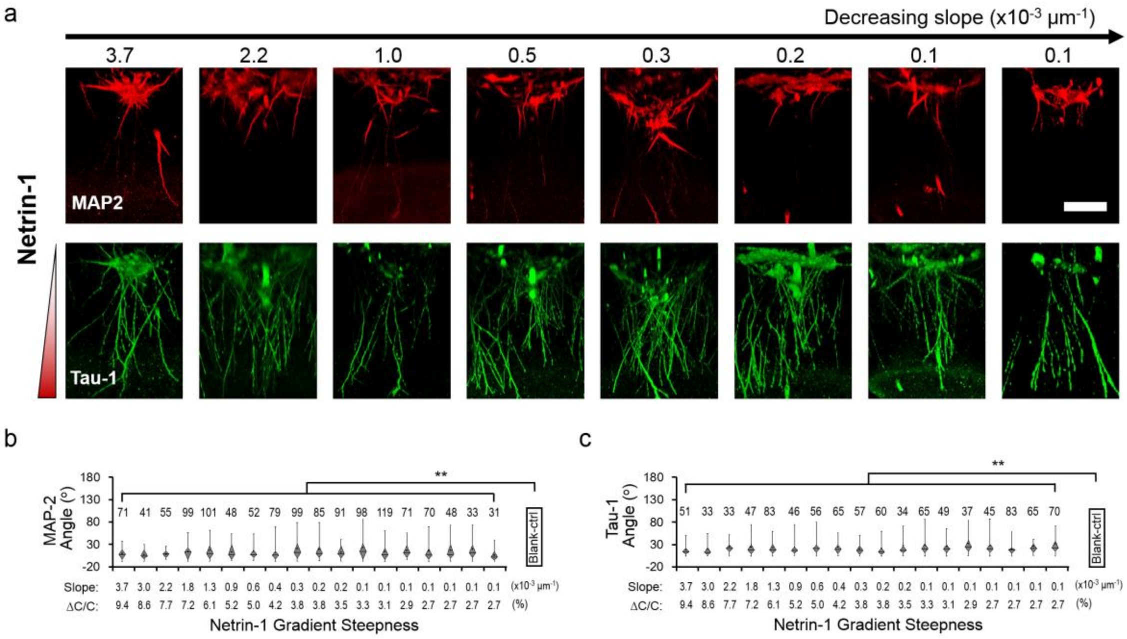

Supplementary Figure 7. Neuronal differentiation in response to netrin-1 gradient with varied steepness at 24 hours. (**a**) Side views of axonal (Tau-1$^+$) or dendritic (MAP2$^+$) differentiation under netrin-1 gradient of decreasing steepness, scale bar, 100 μm. (**b, c**) Box-plots for quantitative analysis of MAP2$^+$ or Tau-1$^+$ neurite guidance in response to netrin-1 gradient of varied steepness. The parts of the box indicate 25, 50 and 75 percentiles, and the whiskers indicate 10 and 90%. The square mark indicates mean of the data. More than 30 neurites (as indicated on top of each box) were pooled from 4 biological replicates. Neuronal growth pattern in each hydrogel cylinder was compared in pairwise to experiments without any chemotactic factor treatment (Blank-ctrl, Supplementary Fig. 6), ** indicates a *p*-value < 0.005 by paired Kruskal-Wallis test.

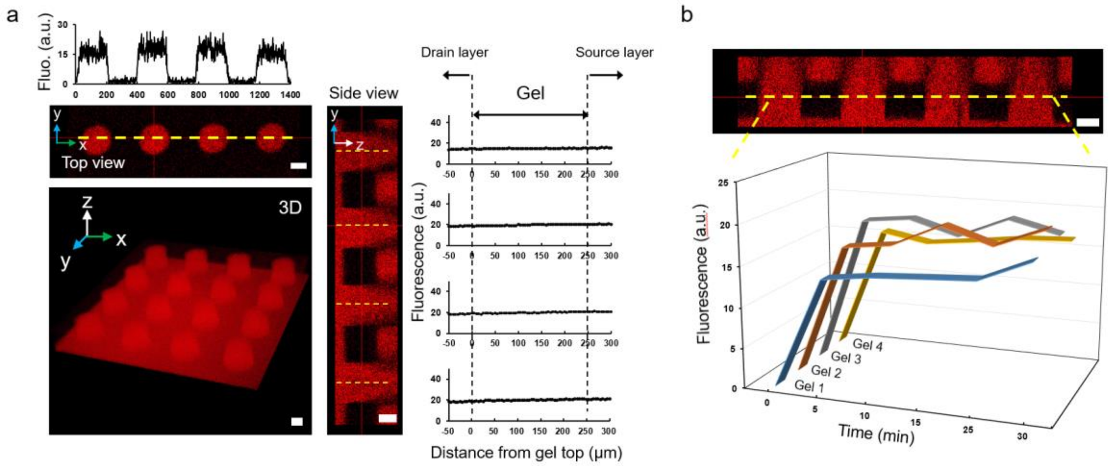

Supplementary Figure 8. The distribution of small molecules in the HT-ChemoChip for efficient drug treatment. (**a**) 3D reconstruction of the confocal scans showing the distribution of fluorescent molecules (Alexa 488, MW 643) in the hydrogel cylinders. The horizontal molecular distribution is shown in the X-Y top view, and the fluorescence variation across the yellow dash line is given in the graph above; the vertical molecular distribution is shown in the Y-Z side view, and the fluorescence variation across the yellow dash lines is given in the graph on the right. (**b**) The temporal change of fluorescence intensity in the hydrogel cylinders over the initial 30 minutes after applying Alexa 488 molecules to a HT-ChemoChip. Scale bar, 100um.

Supplementary Table

Supplementary Table 1. Summary of *p-values* for the statistical analysis of steepness-dependent neuronal chemotactic regulation by Sema3A, NGF or netrin-1 gradient.

Chemotactic Molecular	Treatment	Cellular program	*p*-value	Steepness dependence
Sema3A gradient	24 hours	Cell migration	6.58×10^{-7}	✓
		Neurite repellence	1.11×10^{-13}	✓
		Tau-1^{+} repellence	3.45×10^{-12}	✓
		MAP2^{+} repellence	0.056	
	STK11 knockdown	Cell migration	1.38×10^{-11}	✓
		Neurite repellence	0.876	
	GSK3 inhibition	Cell migration	0.032	
		Neurite repellence	1.39×10^{-6}	✓
	Heat deactivated	Cell migration	0.265	
		Neurite repellence	0.798	
Sema3A, single gradient	24hours	Cell migration	0.200	
		Neurite repellence	0.447	
Sema3A, homogeneous	24 hours	Cell migration	0.070	
		Neurite guidance	0.024	
NGF gradient	24 hours	Cell migration	2.86×10^{-5}	✓
		Neurite guidance	0.973	
Netrin-1 gradient	6 hours	Cell migration	0.197	
		Neurite length	1.21×10^{-3}	✓
Netrin-1 gradient	24 hours	Cell migration	0.351	
		Neurite guidance	0.460	
		Neurite length	0.226	
		Tau-1^{+} guidance	0.030	
		MAP2^{+} guidance	0.061	

Supplementary References

1. Braga, J., Desterro, J. M., Carmo-Fonseca, M. Intracellular macromolecular mobility measured by fluorescence recovery after photobleaching with confocal laser scanning microscopes. *Mol Biol Cell* 15, 4749-4760 (2004).
2. Gribbon, P., Hardingham, T. E. Macromolecular diffusion of biological polymers measured by confocal fluorescence recovery after photobleaching. *Biophys J* 75, 1032-1039 (1998).
3. Altenberger, A. R., Tirrell, M., Dahler, J. S. Hydrodynamic Screening and Particle Dynamics in Porous-Media, Semidilute Polymer-Solutions and Polymer Gels. *J Chem Phys*, 84, 5122-5130 (1986).
4. Phillies, G. D. J. The Hydrodynamic Scaling Model for Polymer Self-Diffusion. *J Phys Chem*, 93, 5029-5039 (1989).
5. Banks, D. S., Fradin, C. Anomalous diffusion of proteins due to molecular crowding. *Biophys J* 89, 2960-2971 (2005).
