# Supplementary material for: High-throughput three-dimensional chemotactic assays reveal steepness-dependent complexity in neuronal sensation to molecular gradients
Source: Nat Commun. 2018 Nov 12;9:4745. doi: 10.1038/s41467-018-07186-x (PMC6232128; doi:10.1038/s41467-018-07186-x)
Supplement: Supplementary file 2 — Description of Additional Supplementary Files [file 41467_2018_7186_MOESM2_ESM.pdf]

Description of Additional Supplementary Files

File Name: Supplementary Movie 1
Description: 3D culture of hippocampal neurons in a hydrogel cylinder containing a netrin-1 gradient.
