# Supplementary material for: High-throughput three-dimensional chemotactic assays reveal steepness-dependent complexity in neuronal sensation to molecular gradients
Source: Nat Commun. 2018 Nov 12;9:4745. doi: 10.1038/s41467-018-07186-x (PMC6232128; doi:10.1038/s41467-018-07186-x)
Supplement: Supplementary file 5 — Reporting Summary [file 41467_2018_7186_MOESM5_ESM.pdf]

nature research

Corresponding author(s): Peng Shi

Reporting Summary

Nature Research wishes to improve the reproducibility of the work that we publish. This form provides structure for consistency and transparency in reporting. For further information on Nature Research policies, see Authors & Referees and the Editorial Policy Checklist.

Statistical parameters

When statistical analyses are reported, confirm that the following items are present in the relevant location (e.g. figure legend, table legend, main text, or Methods section).

n/a	Confirmed	
☐	☒	The exact sample size (n) for each experimental group/condition, given as a discrete number and unit of measurement
☐	☒	An indication of whether measurements were taken from distinct samples or whether the same sample was measured repeatedly
☐	☒	The statistical test(s) used AND whether they are one- or two-sided<br>*Only common tests should be described solely by name; describe more complex techniques in the Methods section.*
☐	☒	A description of all covariates tested
☐	☒	A description of any assumptions or corrections, such as tests of normality and adjustment for multiple comparisons
☐	☒	A full description of the statistics including central tendency (e.g. means) or other basic estimates (e.g. regression coefficient) AND variation (e.g. standard deviation) or associated estimates of uncertainty (e.g. confidence intervals)
☐	☒	For null hypothesis testing, the test statistic (e.g. F, t, r) with confidence intervals, effect sizes, degrees of freedom and P value noted<br>*Give P values as exact values whenever suitable.*
☒	☐	For Bayesian analysis, information on the choice of priors and Markov chain Monte Carlo settings
☒	☐	For hierarchical and complex designs, identification of the appropriate level for tests and full reporting of outcomes
☒	☐	Estimates of effect sizes (e.g. Cohen's d, Pearson's r), indicating how they were calculated
☐	☒	Clearly defined error bars<br>*State explicitly what error bars represent (e.g. SD, SE, CI)*

Our web collection on statistics for biologists may be useful.

Software and code

Policy information about availability of computer code

Data collection	Leica Application Suite
Data analysis	Matlab (9.2); ImageJ (1.51u); R (3.4.3).

For manuscripts utilizing custom algorithms or software that are central to the research but not yet described in published literature, software must be made available to editors/reviewers upon request. We strongly encourage code deposition in a community repository (e.g. GitHub). See the Nature Research guidelines for submitting code & software for further information.

Data

Policy information about availability of data

All manuscripts must include a data availability statement. This statement should provide the following information, where applicable:

- Accession codes, unique identifiers, or web links for publicly available datasets
- A list of figures that have associated raw data
- A description of any restrictions on data availability

The data that support the finding of this study is available from the corresponding author upon request.

Field-specific reporting

Please select the best fit for your research. If you are not sure, read the appropriate sections before making your selection.

☒ Life sciences ☐ Behavioural & social sciences ☐ Ecological, evolutionary & environmental sciences

For a reference copy of the document with all sections, see nature.com/authors/policies/ReportingSummary-flat.pdf

Life sciences study design

All studies must disclose on these points even when the disclosure is negative.

Sample size	At each hydrogel location (ranging from 0.3 to 7.9 mm away from the inlet spaced by 0.4 mm), pairwise Kruskal-Wallis test was performed at each hydrogel cylinder location to determine the statistical significance between the experimental conditions and the control groups, $p < 0.05$ indicates a significant difference. For quantitative analysis, at least three biological replicates were used in this study. From each replicate, 2~3 rows of hydrogel cylinders were quantified; in each hydrogel, 5~10 neurons were measured to access the neurite growth and migration level. For Fig. 3, the condition with netrin-1 or NGF gradient was compared to samples without any factor treatment (Blank-control). For Fig. 4, the condition with Sema3A gradient was compared to Blank-control. For Fig. 5 and Fig. 6 the experimental condition (Sema3A gradient & STK11 knockdown, Sema3A gradient & GSK inhibitor) was compared to samples under regular Sema3A gradient (SMA-Norm).
Data exclusions	No sample were excluded from analysis.
Replication	Multiple biological replicates were performed to confirm the experimental observation.
Randomization	No randomization is needed.
Blinding	During analysis of the neuron growth pattern, the operator was blind to the information regarding the location of hydrogel cylinders.

Reporting for specific materials, systems and methods

Materials & experimental systems

n/a	Involved in the study
☒	☐ Unique biological materials
☒	☐ Antibodies
☒	☐ Eukaryotic cell lines
☒	☐ Palaeontology
☐	☒ Animals and other organisms
☒	☐ Human research participants

Methods

n/a	Involved in the study
☒	☐ ChIP-seq
☒	☐ Flow cytometry
☒	☐ MRI-based neuroimaging

Animals and other organisms

Policy information about studies involving animals; ARRIVE guidelines recommended for reporting animal research

Laboratory animals	E18 Sprague Dawley rats
Wild animals	n.a.
Field-collected samples	n.a.
